# Underlying and immediate causes of death in patients with idiopathic pulmonary fibrosis

**DOI:** 10.1186/s12890-018-0642-4

**Published:** 2018-05-11

**Authors:** Miia Kärkkäinen, Hanna Nurmi, Hannu-Pekka Kettunen, Tuomas Selander, Minna Purokivi, Riitta Kaarteenaho

**Affiliations:** 1 Division of Respiratory Medicine, Institute of Clinical Medicine, School of Medicine, Faculty of Health Sciences, University of Eastern Finland, P.O. Box 1627, 70211 Kuopio, Finland; 2 Kuopio City Home Care, Rehabilitation and Medical Services for Elderly, Tulliportinkatu 37E, 70100 Kuopio, Finland; 3 Center of Medicine and Clinical Research, Division of Respiratory Medicine, Kuopio University Hospital, P.O. Box 100, 70029 KYS Kuopio, Finland; 4 Department of Clinical Radiology, Kuopio University Hospital, P.O. Box 100, 70029 KYS Kuopio, Finland; 5 Science Services Center, Kuopio University Hospital, P.O. Box 100, 70029 KYS Kuopio, Finland; 6 Respiratory Medicine, Research Unit of Internal Medicine, University of Oulu and Medical Research Center Oulu, Oulu University Hospital, P.O. Box 20, 90029 Oulu, Finland; 7 Kuopio University Hospital and University of Eastern Finland, Puijonlaaksontie 2, 70210, P.O. Box 100, 70029 KYS Kuopio, Finland

**Keywords:** Cause of death, Idiopathic pulmonary fibrosis, Mortality, Acute exacerbation

## Abstract

**Background:**

The most common cause of death of patients with idiopathic pulmonary fibrosis (IPF) has been reported to be the lung disease itself and mortality from IPF appears to be increasing. However, the causes of death in patients with IPF taking into account differences between genders and smoking histories as well as disease progression, have not been previously explored.

**Methods:**

Retrospective data from hospital register and death certificates from national database of IPF patients treated in Kuopio University Hospital (KUH) from 2002 to 2012 were collected. Mortality was also explored from the death registry database via ICD-10 code J84 revealing the numbers of deaths from pulmonary fibrosis in Finland from 1998 to 2015.

**Results:**

Out of 117 deaths, 26.5% were females and 73.5% males in KUH. The most common underlying causes of death were IPF 67.5% and ischemic heart diseases 14.8%. More males died for reasons other than IPF (39.5%) compared to females (12.9%) (*p* = 0.007). Pneumonia as the immediate cause of death was more common in males (27.9%) than in females (3.2%) (*p* = 0.004) and in ex-smokers (32.7%) compared to non-smokers (9.3%) (*p* = 0.007). Death register based mortality from pulmonary fibrosis is increasing in Finland.

**Conclusions:**

Even though the overall mortality was higher in males with IPF, the disease-specific mortality for IPF was higher in females i.e. in males, comorbidities were more often the underlying causes of death. Pneumonia-triggered acute exacerbations of IPF may be associated with smoking and gender since females and non-smokers were less likely to succumb to pneumonia. We conclude that disease progression at the end of life may vary depending on smoking habits and gender.

## Background

Idiopathic pulmonary fibrosis (IPF) is the most common form of the idiopathic interstitial lung diseases [1]. The most common cause of death in IPF patients has been reported to be the disease itself followed by cardiac disorders and lung cancer [2–5]. A rapid deterioration of the disease may be caused by pulmonary embolism, pneumothorax, infections or heart failure [6]. Nearly half of all patients (47%) dying primarily from IPF had experienced a deterioration of their lung disease prior to their death [7]. A new definition and revised diagnostic criteria for acute exacerbation of IPF have been proposed recently; exclusion of infection or other potential triggers is no longer required, the only qualifier is that the clinician should determine that cardiac failure or fluid overload does not fully explain the radiological findings of new bilateral ground glass opacification or consolidation [8].

Global mortality from IPF has been increasing, with males being reported to have higher mortality than females [9–12]. It seems that mortality varies between countries depending on the definition and diagnosis codes used [9]. In the years 2011–2013 in the European Union (EU), the highest IPF mortality rates were reported in the United Kingdom while the lowest rates were in Lithuania [12]. In Finland, the median mortality rate for males was 7.36 per 100,000 and 3.62 per 100,000 for females, these values being the second highest in the EU [12].

The aim of this study was to examine the mortality rates of patients with IPF in the Kuopio University Hospital (KUH) area located in eastern Finland. The underlying and immediate causes of death from the patients’ death certificates were gathered. All data was explored in the whole study group as well as separately for females and males. Smoking history, disease progression categorized according to the life span into rapid (0–2 years), moderate (2–5 years) and slow (> 5 years) as well as gender-age-physiology (GAP) stage were taken into account. Furthermore, the total numbers of deaths due to pulmonary fibrosis (PF) according to the ICD-10 code J84 were investigated in Finland between the years 1998–2015 from the national registry database of Statistics Finland and this data was compared to that from the local area served by KUH.

## Methods

### Patients and data collection

A total of 223 patients (91 female, 132 male) with PF treated in KUH between 1st January 2002 and 31st December 2012 were collected from the hospital’s medical records by using ICD-10 codes J84.1, J84.8 and J84.9. Clinical, radiological and histological information of each patient was transferred from medical records to special forms designed for the present study. PF with a known etiology i.e. associated with rheumatoid arthritis or connective-tissue disease and asbestosis, were excluded. The data concerning causes of death and places of death was updated on 14th December 2016.

Smoking history was assessed such that the individual was defined as a non-smoker, ex-smoker or current smoker. Pulmonary function tests included results of forced vital capacity (FVC), forced expiratory volume in 1 sec (FEV1), FEV1 / FVC ratio and diffusion capacity of carbon monoxide (DLco). Information was collated from histological samples from surgical lung biopsies or autopsy.

Death causes were obtained from the death certificates using ICD-10 codes and registered as underlying and immediate causes of death. Places of death were also registered. The overall death rates due to PF according to ICD-10 code J84 and ischemic heart diseases (ICD-10 codes I21–25) were obtained from the open national database of Statistics Finland, in which the ICD-10 codes are available only at a 3-character level, and therefore data with more specific codes, such as J84.1, was not available “http://www.stat.fi/til/ksyyt/2015/ksyyt_2015_2016-12-30_tau_001_en.html”. In Finland, the disease classification of the death certificates was altered in the years 2005–2006. Subsequently, pneumonia was no longer accepted as the underlying cause of death if some other chronic disease had weakened the patient prior to the development of pneumonia. In such cases, the weakening chronic illness is recorded as the underlying cause of death “http://www.stat.fi/til/ksyyt/2015/ksyyt_2015_2016-12-30_tau_001_en.html”.

Acute exacerbation was defined using diagnostic criteria with 1) a previous diagnosis of IPF 2) acute worsening or development of dyspnea typically of less than 1 month duration 3) computed tomography with new bilateral ground-glass opacity and/or consolidation superimposed on a background pattern consistent with UIP and 4) a deterioration not fully explained by cardiac failure or fluid overload [8]. If computed tomography was not available, the designation of suspected acute exacerbation of IPF was used.

No consents for the inclusion into this study were collected since it was retrospective, with the majority of the patients already deceased. The study protocol was approved by the Research Ethics Committee of the Northern Savo Hospital District (statement 17/2013) and from the National Institute for Health and Welfare (Dnro THL/1052/5.05.01/2013). Permission to use data from death certificates was given by Statistics Finland (Dnro: TK-53-911-13). This study was conducted in compliance with the Declaration of Helsinki.

### Assessment of the data

The findings of the first and last high resolution computed tomography (HRCT) of each patient were re-evaluated by an experienced radiologist according to the recent statement of the American Thoracic Society (ATS) and the European Respiratory Society (ERS) and re-classified as usual interstitial pneumonia (UIP), possible UIP and not UIP [1, 13]. All the patients whose re-analyses were categorized as not definite UIP in HRCT or histology were evaluated during a multidisciplinary discussion with a radiologist, a pathologist and a pulmonologist before inclusion into the present study.

The patients were categorized into classes according to the observed time elapsing until their death: 1) rapid (0–2 years), 2) moderate (2–5 years) and 3) slow (> 5 years). Those patients that were still alive and with a follow-up time less than 5 years were not included into the categorization. The GAP stage was calculated using gender, age and results of FVC and DLco (physiology) at the time of IPF diagnosis to obtain a total points score, which was then used to classify patients into stages I (0–3 points), II (4–5 points), or III (6–8 points) [14].

### Statistical analysis

Statistical analysis was performed with IBM SPSS Statistics version 21. Group differences were tested by Chi-square testing or Fisher exact test with categorical variables. Data is presented as mean with standard deviation for continuous variables or frequencies with percentages when variables are categorical. The survival was estimated by the Kaplan-Meier method using the time of diagnosis as the starting-point and death or lung transplantation as the end-point. The median survival time is presented. Multivariate survival analyses were computed using Cox regression models and results are shown in the form of a hazard ratio (HR) with 95% confidence intervals. *P*-value < 0.05 was considered as statistically significant.

## Results

### Patient characteristics

After re-analyses of radiological, histological and clinical data from a total of 223 patients, 89 cases were excluded due to diagnoses other than IPF, i.e. 132 patients with IPF were included in this study. Four patients had missing data of their smoking histories. Out of 128 patients, 45 (35.2%) were non-smokers, 66 (51.6%) were ex-smokers and 17 (13.3%) were current smokers. A total of 117 deaths had occurred by 14th December 2016. Three male patients, two current smokers and one ex-smoker, had undergone lung transplantation; one of them was a current smoker and had subsequently died. We calculated the following mean ages; the time of diagnosis, 70.5 years (SD 9.78); at death, 74.8 years (SD 8.98) and at lung transplantation, 53.3 years (SD 8.08). At the time of diagnosis, mean FVC was 76.7% predicted (SD 18.51), mean FEV1 77.0% predicted (SD 16.99), mean FEV1/FVC 101.4% predicted (SD 9.68) and mean DLco 56.1% predicted (SD 17.51) (Table 1.)

**Table 1:** Demographic characteristics of the patients

	Total	Male	Female	*p*-value^a^
N (%)	132 (100)	97 (73.5)	35 (26.5)	
Age years (SD)	70.5 (9.78)	70.2 (10.34)	71.3 (8.17)	0.562
Median survival (Mo)	42.0	36.0	60.0	0.045
Non-smokers n (%)	45 (35.2)	21 (22.1)	24 (72.7)	< 0.001
Former smokers n (%)	66 (51.6)	60 (63.2)	6 (18.2)	< 0.001
Current smokers n (%)	17 (13.3)	14 (14.7)	3 (9.1)	< 0.001
*Pulmonary function test results at the time of diagnosis* ^*b*^				
FVC % predicted (SD)	76.7 (18.51)	75.7 (18.91)	79.2 (17.39)	0.354
FEV1% predicted (SD)	77.0 (16.99)	75.3 (17.22)	81.6 (15.66)	0.065
FEV1/FVC % predicted	101.4 (9.68)	100.3 (9.45)	104.2 (9.88)	0.049
DLCO % predicted (SD)	56.1 (17.51)	55.8 (18.02)	56.7 (16.33)	0.803

*p*-values calculated using Fisher test, Chi-squared test or Kaplan-Meier log-rank test

^a^Between genders

^b^Spirometry results were missing from 6 patients and diffusion capacity results from 8 patients

*N* number, *Mo* months, *FVC* forced vital capacity, *FEV1* forced expiratory volume in one second, *DLco* diffusion capacity of carbon monoxide

The median survival was 42.0 months. Fifty (43.5%) patients died in a tertiary hospital; 4 of them (8.0%) in an intensive care unit; 50 (43.5%) in a community hospital, 16 (13.9%) at home and 1 (0.9%) in a nursing home.

### Mortality

**Fig. 1:**
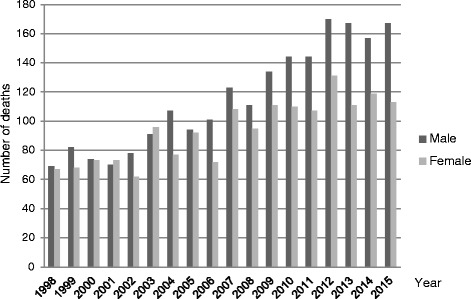
The number of pulmonary fibrosis deaths in Finland between the years 1998–2015 “http://www.stat.fi/til/ksyyt/2015/ksyyt_2015_2016-12-30_tau_001_en.html”. Underlying cause of death according to J84 (ICD-10, 3-character level): The data of this time series is otherwise comparable, but the classification of pneumonia was altered in 2005 and 2006. From that time onwards, an international guideline was adopted. Accordingly, pneumonia is not accepted as the underlying cause of death if a chronic disease which has weakened the individual, is mentioned in the death certificate. In such cases, the disease in question is recorded as the person’s underlying cause of death. The revision of the classification was gradually taken into use in 2005 and 2006 “http://www.stat.fi/til/ksyyt/2015/ksyyt_2015_2016-12-30_tau_001_en.html”

In the KUH district, which was serving 248,000 citizens at the end of year 2012, the 5 year overall mortality rate between the years 2003–2007 was 2.5 per 100,000 whereas between the years 2008–2012, it was 4.7 per 100,000. When including only patients with IPF (ICD-10 code J84.1) as the underlying cause of death i.e. disease-specific mortality, the 5 year mortality rates were 1.7 per 100,000 and 3.1 per 100,000 in 2011 and 2012, respectively. The total numbers of PF deaths (ICD-10 code J84) in Finland from 1998 to 2015 are presented in Fig. 1 “http://www.stat.fi/til/ksyyt/2015/ksyyt_2015_2016-12-30_tau_001_en.html”. There were 280 PF deaths in the year 2015 in Finland; from these values, one can calculate a mortality rate of 5.1 per 100,000, when the official total population of Finland at the end of the year 2015 was 5,487,308 “http://www.stat.fi/til/ksyyt/2015/ksyyt_2015_2016-12-30_tau_001_en.html; http://www.stat.fi/til/vaerak/2015/vaerak_2015_2016-04-01_tie_001_en.html?ad=notify”.

The 5 year mortality rates in KUH district between years 2003–2007 were 0.7 and 1.9 per 100,000 for females and males, respectively; between the years 2008–2012, the mortality rates were 1.85 per 100,000 for females and 3.3 per 100,000 for males.

### Exacerbations and causes of death

The main underlying causes of death were IPF, ischemic heart disease and lung cancer. The most common immediate causes of death were IPF, pneumonia and ischemic heart disease (Table 2). An acute exacerbation was observed in 10 (12.7%) and a suspected acute exacerbation of IPF in 28 (35.4%) patients with IPF as the underlying cause of death. An acute exacerbation was observed in 7 (12.9%) and a suspected acute exacerbation in 18 (33.3%) patients with IPF as the immediate causes of death. Sixty (52.2%) patients had coronary artery disease (CAD) as a comorbidity from which 28.3% had ischemic heart disease as the underlying cause of death and 25.0% as the immediate causes of death. Compared to males, a higher percentage of females died from IPF. In contrast, more males than females had pneumonia as the immediate cause of death (Table 2).

**Table 2:** Causes of death in the patients with IPF in the Kuopio University Hospital area

	Total (*n* = 132)	Female (*n* = 35)	Male (*n* = 97)	*p*-value^a^
Deaths N (%)	117 (88.6)	31 (88.6)	86 (90.7)	0.774
Underlying cause of death				
IPF	79 (67.5)	27 (87.1)	52 (60.5)	0.007
Pneumonia	3 (2.3)		3 (3.1)	0.292
Ischemic heart disease	17 (14.5)	3 (9.7)	14 (16.3)	0.371
Lung cancer	7 (6.0)		7 (8.1)	0.101
Pulmonary embolism	2 (1.7)	1 (3.2)	1 (1.2)	0.447
Colon cancer	2 (1.7)		2 (2.3)	0.392
COPD	1 (0.9)		1 (1.2)	0.547
Other^b^	6 (5.2)		6 (7.1)	
Immediate cause of death				
IPF	54 (47.0)	23 (74.2)	31 (36.0)	< 0.001
Pneumonia	25 (21.4)	1 (3.2)	24 (27.9)	0.004
Ischemic heart disease	15 (12.8)	2 (6.5)	13 (15.1)	0.216
Heart failure^c^	8 (6.8)	2 (6.5)	6 (7.0)	0.921
Lung cancer	5 (4.3)		5 (5.8)	0.170
Pulmonary embolism	2 (1.7)	1 (3.2)	1 (1.2)	0.447
Colon cancer	2 (1.7)		2 (2.4)	0.392
Other^d^	6 (5.2)	1 (3.2)	5 (6.0)	
Exacerbations preceding death				
Acute exacerbation	11 (9.4)	3 (9.7)	8 (9.3)	0.932
Suspected acute exacerbation^e^	31 (26.5)	7 (22.6)	24 (27.9)	0.609

p-values calculated using Fisher test

*IPF* idiopathic pulmonary fibrosis, *COPD* chronic obstructive pulmonary disease;

^a^Between genders

^b^Sick sinus syndrome (*N* = 1), cerebral infarction (*N* = 1), aortic rupture (*N* = 1), ileum strangulation (*N* = 1), heart failure (*N* = 1), drowning (*N* = 1)

^c^For any reason, including cor pulmonale

^d^Cerebral infarction (*N* = 1), aortic arteriosclerosis (*N* = 1), ileum strangulation (*N* = 1), hypotension (*N* = 1), respiratory failure due to COPD (*N* = 1), drowning (*N* = 1)

^e^No HRCT confirmation

Ex-smokers and current smokers had more often lung cancer as the underlying cause of death compared to non-smokers (*p* = 0.045 and 0.016, respectively). Non-smokers had more often IPF and less often pneumonia mentioned as the immediate cause of death compared to ex-smokers (*p* = 0.043 and *p* = 0.007, respectively). A trend towards a statistically significant difference was observed when comparing the underlying causes of death between non-smokers and current smokers (*p* = 0.062 for IPF and *p* = 0.091 for pneumonia). No statistically significant differences were observed in the causes of death between ex-smokers and current smokers (Table 3).

**Table 3:** Causes of death according to smoking history

	Non-smokers (*n* = 45)	Ex-smokers (*n* = 66)	Current smoker (*n* = 17)	*p*-value^a^
Total N of deaths (%)	43 (95.5)	55 (83.3)	15 (88.2)	0.153
Underlying cause of death				
IPF	34 (79.1)	35 (63.6)	8 (53.3)	0.111
Pneumonia		2 (3.6)	1 (6.7)	0.315
Ischemic heart disease	6 (14.0)	6 (10.9)	3 (20.0)	0.646
Lung cancer		5 (9.1)	2 (13.3)	0.084
Pulmonary embolism	2 (4.7)			0.191
Colon cancer		2 (3.6)		0.342
COPD			1 (6.7)	0.133
Other^b^	1 (2.3)	5 (9.1)		
Immediate cause of death				
IPF	27 (60.5)	21 (38.2)	6 (40.0)	0.076
Pneumonia	4 (9.3)	18 (32.7)	3 (20.0)	0.021
Ischemic heart disease	5 (11.6)	6 (10.9)	3 (20.0)	0.627
Heart failure^c^	4 (9.3)	2 (3.6)	1 (6.7)	0.512
Lung cancer		4 (7.3)	1 (6.7)	0.200
Pulmonary embolism	2 (4.8)			0.191
Colon cancer		2 (3.6)		0.342
Other^d^	1 (2.3)	3 (5.5)	1 (7.1)	
Exacerbations preceding death				
Acute exacerbation	5 (11.6)	6 (10.7)		0.370
Suspected acute exacerbation^e^	11 (25.6)	16 (28.6)	4 (25.0)	0.929

p-values calculated using Fisher test

*IPF* idiopathic pulmonary fibrosis, *COPD* chronic obstructive pulmonary disease;

^a^*p*-value between different smoking histories

^b^Sick sinus syndrome (*N* = 1), cerebral infarction (*N* = 1), aortic rupture (*N* = 1), ileum strangulation (*N* = 1), heart failure (*N* = 1), drowning (*N* = 1)

^c^For any reason, including cor pulmonale

^d^Cerebral infarction (*N* = 1), aortic arteriosclerosis (*N* = 1), ileum strangulation (N = 1), hypotension (*N* = 1), respiratory failure due to COPD (*N* = 1), drowning (*N* = 1)

^e^No HRCT confirmation

A multivariate analysis was performed to investigate whether differences in deaths between genders could potentially be explained by smoking status or vice versa. It was found that both gender and smoking status were independent predictors of mortality; for males, the HR was 1.7 (1.05–2.90) compared to females (*p* = 0.033) and for ex-smokers, the HR was 2.0 (1.10–3.60) compared to current smokers (*p* = 0.024).

There were significantly more deaths (100%) in the rapid disease progression group compared to slow disease progression (80%) (*p* = 0.006). More patients with a slow disease progression had IPF as the underlying cause of death compared to patients with a moderate disease progression (*p* = 0.033). More patients with a rapid disease progression died from an acute exacerbation compared to their counterparts with a moderate and slow disease progression (Table 4). No statistically significant differences were detected in the causes of death in the different GAP stages (Table 5).

**Table 4:** Causes of death in different types of disease progression

	Rapid (*n* = 40)	Moderate (*n* = 42)	Slow (*n* = 45)	*p*-value^a^
Total N of deaths (%)	40 (100)	41 (97.6)	36 (80.0)	< 0.001
Underlying cause of death				
IPF	29 (72.5)	22 (53.7)	28 (77.8)	0.056
Pneumonia	2 (5.0)	1 (2.4)		0.387
Ischemic heart disease	5 (12.5)	9 (22.0)	3 (8.3)	0.216
Lung cancer	1 (2.5)	4 (9.8)	2 (5.6)	0.384
Pulmonary embolism	1 (2.5)		1 (2.8)	0.575
Colon cancer		1 (2.4)	1 (2.8)	0.586
COPD		1 (2.4)		0.393
Other^b^	1 (2.0)	3 (4.9)	1 (2.8)	
Immediate cause of death				
IPF	20 (50.0)	16 (39.0)	18 (50.0)	0.524
Pneumonia	10 (25.0)	9 (22.0)	6 (16.7)	0.672
Ischemic heart disease	5 (12.5)	7 (17.1)	3 (8.3)	0.518
Heart failure^c^	1 (2.5)	3 (7.3)	4 (11.1)	0.328
Lung cancer	1 (2.5)	2 (4.9)	2 (5.6)	0.783
Pulmonary embolism	1 (2.5)		1 (2.8)	0.575
Colon cancer		1 (2.4)	1 (2.8)	0.586
Other^d^	2 (4.1)	3 (7.3)	1 (2.8)	
Exacerbations preceding death				
Acute exacerbation	8 (20.0)	1 (2.4)	2 (5.4)	0.014
Suspected acute exacerbation^e^	15 (37.5)	6 (14.3)	10 (27.0)	0.056

p-values calculated using Fisher test

Rapid = survival ≤2 years, moderate = survival 2–5 years, slow = survival ≥5 years

*IPF* idiopathic pulmonary fibrosis, *COPD* chronic obstructive pulmonary disease

^a^*p*-value between the groups of different disease progression

^b^Sick sinus syndrome (*N* = 1), cerebral infarction (*N* = 1), aortic rupture (*N* = 1), ileum strangulation (*N* = 1), heart failure (*N* = 1), drowning (*N* = 1)

^c^For any reason, including cor pulmonale

^d^Cerebral infarction (*N* = 1), aortic arteriosclerosis (*N* = 1), ileum strangulation (*N* = 1), hypotension (*N* = 1), respiratory failure due to COPD (*N* = 1), drowning (*N* = 1)

^e^No HRCT confirmation

**Table 5:** Causes of death according to the GAP stage

	GAP I (*n* = 67)	GAP II (*n* = 47)	GAP III (*n* = 12)	*p*-value^a^
Deaths N (%)	57 (85.1)	42 (93.3)	12 (100)	0.455
Underlying cause of death				
IPF	41 (71.9)	24 (57.1)	10 (83.3)	0.140
Pneumonia		3 (7.1)		0.079
Ischemic heart disease	10 (17.5)	6 (14.3)	1 (8.3)	0.703
Lung cancer	3 (5.3)	3 (7.1)	1 (8.3)	0.888
Pulmonary embolism	1 (1.8)			0.620
Colon cancer	2 (3.5)			0.381
COPD		1 (2.4)		0.437
Other^b^		5 (11.9)		
Immediate cause of death				
IPF	28 (49.1)	17 (40.5)	7 (58.3)	0.487
Pneumonia	10 (17.5)	11 (26.2)	3 (25.0)	0.561
Ischemic heart disease	3 (5.3)	3 (7.1)		0.440
Heart failure^c^	4 (7.1)	3 (7.1)		0.636
Lung cancer	2 (3.5)	2 (4.8)	1 (8.3)	0.761
Pulmonary embolism	1 (1.8)			0.620
Colon cancer	2 (3.5)			0.381
Other^d^		4 (9.5)		
Exacerbations preceding death				
Acute exacerbation	6 (10.2)	3 (7.1)	2 (16.7)	0.610
Suspected acute exacerbation^e^	10 (16.9)	14 (33.3)	5 (41.7)	0.072

p-values calculated using Fisher test

*GAP* Gender-Age-Physiology stage, *IPF* idiopathic pulmonary fibrosis, *COPD* chronic obstructive pulmonary disease

^a^*p*-value between different GAP-stages

^b^Sick sinus syndrome (*N* = 1), cerebral infarction (*N* = 1), aortic rupture (*N* = 1), ileum strangulation (*N* = 1), heart failure (*N* = 1), drowning (*N* = 1)

^c^For any reason, including cor pulmonale; ^d^ Cerebral infarction (*N* = 1), aortic arteriosclerosis (*N* = 1), ileum strangulation (*N* = 1), hypotension (*N* = 1), respiratory failure due to COPD (*N* = 1), drowning (*N* = 1)

^e^No HRCT confirmation

## Discussion

As far as we are aware, this is the first detailed clarification of the causes of death in patients with IPF taking into account differences between genders and smoking histories as well as disease progression. Even in this relatively small patient cohort, differences in the causes of death were detected between females and males. Moreover, by examining the smoking histories, we observed that more males and ex-smokers as well as more current smokers died of triggered exacerbations of IPF. Furthermore, more patients with a rapid disease progression died from an acute exacerbation compared to the other disease progression subtypes.

Depending on the methods and diagnostic codes used, the mortality rates have been shown to vary globally and in different countries in the EU [9, 12]. Data from death certificates using ICD-10 codes J84 mortality in 2012 have been reported to be 4.25 per 100,000 in Sweden, 8.88 per 100,000 in Scotland and 8.22 per 100,000 in England and Wales [15]. In Finland, the mortality rate was 5.1 per 100,000 in 2012, being rather similar to the value from Sweden. When classified according to code J84.1, mortality in England and Wales was reported to be 6.90 per 100,000 in 2012 which is higher than our value i.e. 4.7 per 100,000 in the period from 2008 to 2012 [15]. A recent publication based on World Health Organization (WHO) mortality data revealed that the median IPF mortality rate has been rising between the years 2001 and 2013 in 10 out of 17 EU countries, being highest in the United Kingdom (12.01 and 5.63 per 100,000 for males and females, respectively) [12]. The mortality rates in Finland were the second highest i.e. 7.36 and 3.62 per 100,000 for males and females, respectively [12]. Our results are in line with the above-mentioned study revealing that mortality of PF and IPF has risen during the twenty-first century in Finland [12].

The number of PF deaths in Finland has doubled in the last 15 years; in addition, a gender difference has become more apparent since currently more males than females die from PF “http://www.stat.fi/til/ksyyt/2015/ksyyt_2015_2016-12-30_tau_001_en.html”. It could be argued that the changes in the death certificates’ disease classification in the years 2005–2006 may have had some effect on the rising number of PF deaths in Finland “http://www.stat.fi/til/ksyyt/2015/ksyyt_2015_2016-12-30_tau_001_en.html”. This argument, however, was not supported by the results of our study on KUH patient cohort, since the numbers of the patients with pneumonia as the underlying cause of death have not changed after the year 2006; in fact, only three patients had pneumonia as the underlying cause of death during the complete study period. However, the diagnostic methods have improved in the past 10–20 years after the appearance of ATS guidelines for IPF in 2000 [16]. It is possible that more of the other types of interstitial lung diseases such as non-specific interstitial pneumonia were included with IPF at the beginning of the twenty-first century. Since IPF is the most common form of idiopathic PF, the numbers of deaths for PF coded as J84 are likely to reflect the actual number of IPF deaths. The rising trend was also seen in the mortality rate in the KUH cohort. This proposal is supported by the recent data from the WHO mortality database, from which it can be estimated that in the year 2001 the mortality rates in males were 4.7 and 5.0 per 100,000 for the narrow (ICD-10 code J84.1) and the broad (ICD-10 code J84) definitions, respectively, while in females, the rates were around 3.0 per 100,000 with both definitions [12]. In the year 2013, the estimated mortality rates were 7.9 and 8.6 per 100,000 for males, and 3.5 and 3.9 per 100,000 for females, with the narrow and broad definitions, respectively [12]. Thus, it can be concluded that most of the cases coded as J84 represented code J84.1. Moreover, since 30% of IPF patients in the KUH area died for reasons other than IPF, one can estimate from the statistics that approximately 350–400 IPF patients die every year in Finland.

A previous study reported that most IPF patients with CAD die from IPF and only 12% from CAD [17]. Here, the number of ischemic heart disease deaths among IPF patients with CAD was higher (28.3%) probably due to the large disease burden of CAD and other cardiovascular diseases in Finland “http://urn.fi/URN:NBN:fi-fe2016111829194”. However, the total proportion of ischemic heart disease deaths was in line with published reports [3, 5]. In our previous study, we determined that 72.7% of the patients had one or more cardiovascular diseases (hypertension, CAD, cerebral infarction) as a comorbidity and CAD was diagnosed in 49.2% of the patients [13]. Lung cancer accounted for approximately 6% of the deaths in this study, which is somewhat less common than in the previous studies [4, 18].

One can speculate on the reasons for the relatively high number of pneumonias and the low number of acute exacerbations. The data of the current study was collected from the IPF patients diagnosed in KUH between the years 2002–2012 i.e. during the period when the first international perspectives including diagnostic criteria for acute exacerbation of IPF were published (2007) [19]. Since then, Finnish physicians specializing in respiratory medicine have gradually become aware of the exacerbation phenomenon. Since nearly 60% of the patients in our study died outside of tertiary centers, most of the death certificates had been prepared by primary health care physicians. Thus, it is possible that the acute exacerbations were not correctly identified due to the time point of the study and the places of death. Furthermore, HRCT, which would have been needed for diagnosing an acute exacerbation of IPF, is usually not available in the community hospitals in which most of the deaths occurred.

Never-smokers have been claimed to possess a higher risk for acute exacerbation of IPF, although this finding has not been confirmed in all studies [20–22]. A recent study revealed no gender difference in the numbers of acute exacerbations [22]. We observed that few death certificates reported acute exacerbations whereas pneumonias were reported to be the second most common immediate cause of death. Pneumonias are likely to represent triggered acute exacerbations as defined in the recent statement [8]. Since we found significantly less mentions of pneumonia as the immediate cause of death in females than in males and in non-smokers than ex-smokers, it seems that males and ex-smokers experienced more acute pneumonia-triggered exacerbation-linked deaths than females and non-smokers. If that is the case, the exacerbation may be associated with smoking since non-smokers were also less likely to suffer pneumonia than current smokers.

A recently published study, which investigated causes of death associated with GAP scoring (using GAP points), detected no differences between the groups with different scores [23]. In our study, more patients in GAP stage III (GAP score 6) died from IPF (83.0% vs 46.2%) and fewer from infections (0 vs 30.8%). The other GAP stages are more difficult to compare due to their different study approach. However, our study confirms that acute exacerbation leading to death may develop at any stage of the disease. We also found that IPF patients living less than 2 years suffered from lethal acute exacerbations more often than patients with longer survival times.

The limitations of the study mainly result from the relatively small number of patients and the retrospective study protocol. Our data is, however, comparable with those of other studies and moreover, it was very thoroughly collected and re-evaluated. Since histological data was available from only a minority of patients, the diagnoses are mostly based on clinical and radiological investigations which reflect diagnostic procedures of true clinical practice. The percentage of histological diagnoses in this study, however, was comparable with most of the previous studies. Nevertheless, the radiological data of all patients has been carefully re-evaluated and re-classified. The decision to utilize the national death registry base of Finland and its ICD-10 code at the 3 character level i.e. J84, may mean that it includes other fibrotic lung diseases in addition to IPF. We aimed to reduce this potential inaccuracy by investigating the same ICD-codes in our local KUH cohort.

## Conclusions

The mortality determined in this study population reflects the demographics of IPF since there has been a year-on-year increase in the numbers of males and smokers with IPF dying from this disease. Even though the overall mortality was higher in males, the disease-specific mortality for IPF was higher in females, with males having more commonly comorbidities as the underlying cause of death. An acute exacerbation of IPF may be associated with smoking and gender since females and non-smokers were less likely to develop pneumonia, an illness that is known to trigger acute exacerbations. Thus, we conclude that disease progression at the end of life may vary depending on smoking habits and gender in IPF patients.

## Abbreviations

ATS: American Thoracic Society
CAD: Coronary artery disease
DLco: Diffusion capacity of carbon monoxide
ERS: European Respiratory Society
EU: European Union
FEV1: Forced expiratory volume in one second
FVC: Forced vital capacity
GAP: Gender-age-physiology index
HR: Hazard ratio
HRCT: High resolution computed tomography
ICD-10: International Classification of Diseases version 10
IPF: Idiopathic pulmonary fibrosis
KUH: Kuopio University Hospital
PF: Pulmonary fibrosis
UIP: Usual interstitial pneumonia
WHO: World Health Organization

## References

[1] Raghu G, Collard HR, Egan JJ, Martinez FJ, Behr J, Brown KK (2011). An official ATS/ERS/JRS/ALAT statement: idiopathic pulmonary fibrosis: evidence-based guidelines for diagnosis and management. Am J Respir Crit Care Med.

[2] Mannino DM, Etzel RA, Parrish RG (1996). Pulmonary fibrosis deaths in the United States, 1979-1991. An analysis of multiple-cause mortality data. Am J Respir Crit Care Med.

[3] King TE, Albera C, Bradford WZ, Costabel U, du Bois RM, Leff JA (2014). All-cause mortality rate in patients with idiopathic pulmonary fibrosis. Implications for the design and execution of clinical trials. Am J Respir Crit Care Med.

[4] Kreuter M, Ehlers-Tenenbaum S, Palmowski K, Bruhwyler J, Oltmanns U, Muley T (2016). Impact of comorbidities on mortality in patients with idiopathic pulmonary fibrosis. PLoS One.

[5] Rajala K, Lehto JT, Saarinen M, Sutinen E, Saarto T, Myllarniemi M (2016). End-of-life care of patients with idiopathic pulmonary fibrosis. BMC Palliat Care.

[6] Panos RJ, Mortenson RL, Niccoli SA, King TE (1990). Clinical deterioration in patients with idiopathic pulmonary fibrosis: causes and assessment. Am J Med.

[7] Martinez FJ, Safrin S, Weycker D, Starko KM, Bradford WZ, King TE (2005). The clinical course of patients with idiopathic pulmonary fibrosis. Ann Intern Med.

[8] Collard HR, Ryerson CJ, Corte TJ, Jenkins G, Kondoh Y, Lederer DJ (2016). Acute exacerbation of idiopathic pulmonary fibrosis. An international working group report. Am J Respir Crit Care Med.

[9] Hutchinson J, Fogarty A, Hubbard R, McKeever T (2015). Global incidence and mortality of idiopathic pulmonary fibrosis: a systematic review. Eur Respir J.

[10] Swigris JJ, Olson AL, Huie TJ, Fernandez-Perez ER, Solomon J, Sprunger D (2012). Ethnic and racial differences in the presence of idiopathic pulmonary fibrosis at death. Respir Med.

[11] Olson AL, Swigris JJ, Lezotte DC, Norris JM, Wilson CG, Brown KK (2007). Mortality from pulmonary fibrosis increased in the United States from 1992 to 2003. Am J Respir Crit Care Med.

[12] Marshall DC, Salciccioli JD, Shea BS, Akuthota P. Trends in mortality from idiopathic pulmonary fibrosis in the European Union: an observational study of the WHO mortality database from 2001-2013. Eur Respir J. 2018;51(1) 10.1183/13993003.01603-2017. Print 2018 Jan 10.1183/13993003.01603-2017 29348182

[13] Karkkainen M, Kettunen HP, Nurmi H, Selander T, Purokivi M, Kaarteenaho R (2017). Effect of smoking and comorbidities on survival in idiopathic pulmonary fibrosis. Respir Res.

[14] Ley B, Ryerson CJ, Vittinghoff E, Ryu JH, Tomassetti S, Lee JS (2012). A multidimensional index and staging system for idiopathic pulmonary fibrosis. Ann Intern Med.

[15] Hutchinson JP, McKeever TM, Fogarty AW, Navaratnam V, Hubbard RB (2014). Increasing g lobal mortality from idiopathic pulmonary fibrosis in the twenty-first century. Ann Am Thorac Soc.

[16] American Thoracic Society (2000). Idiopathic pulmonary fibrosis: diagnosis and treatment. International consensus statement. American Thoracic Society (ATS), and the European Respiratory Society (ERS). Am J Respir Crit Care Med.

[17] Kim WY, Mok Y, Kim GW, Baek SJ, Yun YD, Jee SH (2015). Association between idiopathic pulmonary fibrosis and coronary artery disease: a case-control study and cohort analysis. Sarcoidosis Vasc Diffuse Lung Dis.

[18] Lee SH, Kim SY, Kim DS, Kim YW, Chung MP, Uh ST (2016). Comparisons of prognosis between surgically and clinically diagnosed idiopathic pulmonary fibrosis using gap model: a Korean National Cohort Study. Medicine (Baltimore).

[19] Collard HR, Moore BB, Flaherty KR, Brown KK, Kaner RJ, King TE (2007). Acute exacerbations of idiopathic pulmonary fibrosis. Am J Respir Crit Care Med.

[20] Song JW, Hong SB, Lim CM, Koh Y, Kim DS (2011). Acute exacerbation of idiopathic pulmonary fibrosis: incidence, risk factors and outcome. Eur Respir J.

[21] Kishaba T, Nagano H, Nei Y, Yamashiro S (2016). Clinical characteristics of idiopathic pulmonary fibrosis patients according to their smoking status. J Thorac Dis.

[22] Johannson KA, Vittinghoff E, Lee K, Balmes JR, Ji W, Kaplan GG (2014). Acute exacerbation of idiopathic pulmonary fibrosis associated with air pollution exposure. Eur Respir J.

[23] Lee SH, Kim SY, Kim DS, Kim YW, Chung MP, Uh ST (2016). Predicting survival of patients with idiopathic pulmonary fibrosis using GAP score: a nationwide cohort study. Respir Res.

